# Validity and Reliability of a Noninvasive Device for Measuring Laryngeal Movement During Swallowing

**DOI:** 10.7759/cureus.78873

**Published:** 2025-02-11

**Authors:** Fumitaka Omori, Masako Fujiu-Kurchi, Aya Hirata, Takafumi Yamano

**Affiliations:** 1 Department of Otorhinolaryngology, Fukuoka Dental College Hospital, Fukuoka, JPN; 2 Department of Speech, Language and Hearing Sciences, International University of Health and Welfare, Narita, JPN; 3 Department of Speech, Language and Hearing Sciences, International University of Health and Welfare, Otawara, JPN; 4 Section of Otorhinolaryngology, Department of Medicine, Fukuoka Dental College, Fukuoka, JPN

**Keywords:** deglutition, larynx, medical devices, movement, noninvasive, swallowing

## Abstract

Background

Laryngeal movement plays a crucial role in the process of swallowing. Peak velocity of laryngeal movement has been shown to be useful in predicting laryngeal penetration and aspiration. There is a growing need to accurately capture and quantify laryngeal movements during swallowing.

Objective

This research comprises two investigations that aim to verify and establish the reliability of a noninvasive device designed to measure laryngeal movement during swallowing.

Methods

In Step 1, one healthy male in his 30s underwent simultaneous videofluoroscopy (VF) examination and noninvasive device measurement. VF videos were employed to generate laryngeal movement curves using video analysis software. In Step 2, 19 healthy young adult men (20-39 years old) and 19 healthy older men (60-79 years old) were instructed to perform five saliva swallows each, with a noninvasive device placed against their necks.

Results

In Step 1, the comparison of laryngeal movement curves between VF and the noninvasive device exhibited almost perfect agreement for upward-downward movement, with less agreement noted for anterior-posterior movement. In Step 2, the reliability of the measurements of peak velocity and distance traveled in Nodomiru showed moderate to high reliability for single measurements (intraclass correlation coefficient (ICC) (1,1) = 0.598-0.855) and high reliability for the average of five measurements (ICC (1,5) = 0.694-0.967).

Conclusions

The results of this study suggest that it is possible to measure the upward-downward movement of the larynx in healthy men using a noninvasive device.

## Introduction

Laryngeal movement plays a crucial role in swallowing, involving the closure of the larynx through anterior and superior elevation, coupled with the inversion of the epiglottis. This movement serves as a visual indicator confirming the occurrence of swallowing. Additionally, the peak velocity of laryngeal movement decreases with more severe dysphagia [1]; therefore, it is useful for predicting laryngeal penetration and aspiration [2].

Traditionally, laryngeal movement has been primarily assessed through videofluoroscopy (VF) examination, with subsequent image analysis performed using video analysis software [3-11]. However, challenges arise in VF-based laryngeal movement measurements due to radiation exposure limiting the number of assessments and the inability to promptly assess the quality of laryngeal movement owing to the time-consuming nature of the analysis. Therefore, attempts have been made to noninvasively measure laryngeal movement during swallowing [12,13]. Devices employing piezoelectric pressure sensors [14,15] have shown promise in accurately evaluating laryngeal elevation time, even in subjects with less obvious laryngeal features. Nevertheless, the limitations of such devices include the inability to separately measure laryngeal movement along the vertical and anterior-posterior axes and the lack of real-time laryngeal movement distance measurement.

The B4S™ uses a highly resilient strain gauge sensor to detect the magnitude of elongation [13]. It is employed to measure the vertical movement of the larynx, although it lacks an algorithm to precisely measure laryngeal movement distance. Nodomiru (OE-NDMR01®, Oisaka Electronic, Fukuyama, Japan) is a noninvasive laryngeal movement measurement device developed and manufactured by Haji [16]. This device captures laryngeal prominence movement in the anterior-posterior and vertical directions by placing a photoelectric distance sensor near the front of the neck, detecting the skin’s shape around the larynx. In a previous study, we reported on the characteristics of laryngeal movement distance, peak velocity, and curves during swallowing maneuvers using Nodomiru [17]. However, the validity of simultaneous measurement with VF has not been verified to date.

This study aimed to establish the validity of laryngeal movement curves generated using the Nodomiru compared with VF and assess intrarater reliability in healthy men. The hypotheses of this study are as follows: (1) laryngeal movement curves obtained using VF and the Nodomiru are consistent; and (2) high intrarater reliability of peak velocity and laryngeal movement distance measurements is achieved using the Nodomiru.

## Materials and methods

Nodomiru

**Figure 1:**
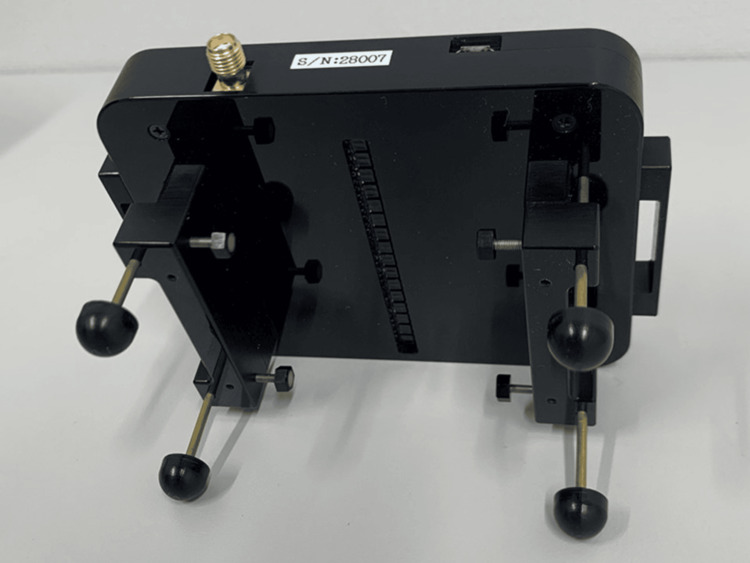
Nodomiru (OE-NDMR01®, Oisaka Electronic, Fukuyama, Japan) The sensor array in the center is used longitudinally in the midline of the neck, relative to the larynx. The wire is connected to a laptop.

Nodomiru, a noninvasive laryngeal elevation measurement system (Nodomiru Application Version 1.07, Oisaka Electronic, Figure 1), was used to measure laryngeal elevation movements. The system consists of two main components: a measurement module that detects skin shape near the larynx, featuring 16 vertically aligned photoelectric distance sensors at 4 mm intervals relative to the front of the neck, and software that captures the sensor information every 0.01 seconds, identifies the larynx, and analyzes and displays its movement.

Alongside the photoelectric distance sensor, the measurement module houses a control CPU, an A/D converter, and a communication circuit. Output information from the sensor is rapidly digitized and transferred to a PC for processing and calculation, enabling real-time data confirmation and storage. The photoelectric distance sensor uses near-infrared light and can operate indoors without light shielding. The instrument module connects via USB to a commercially available Windows PC with installed software [16].

Although Nodomiru can measure anterior-posterior and upward-downward laryngeal movement, a previous study [16] validated reproducibility only for upward-downward movement. Additionally, Nodomiru automatically applies a smoothing process with moving averages for each of the three datasets. The smoothing process is a method for removing unnecessary noise and outliers from data and estimating a smooth true value.

Step 1: Validity of the Laryngeal Movement Curve Generated Using Nodomiru

Participant: The participants included one male in his 30s, a hospital staff member with no history of dysphagia-causing diseases. He was 180 cm tall and 65 kg, and his larynx was easily visible.

**Figure 2:**
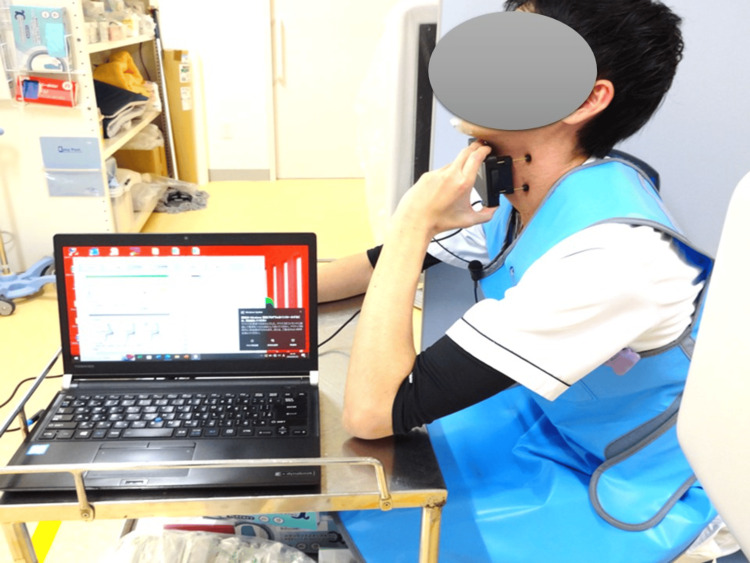
Measurement during Step 1 The participant held the instrument with one hand and assumed a posture that made the larynx visible by guiding their gaze slightly upward. To minimize head and neck movements, the instrument was pressed against the neck with the elbow resting on a desk. A lead ball (11 mm in diameter) was taped to the participant’s lower jaw, and they were seated in front of a fluoroscopy device (DREX-U180, CANON, Tokyo, Japan) while wearing protective gear.

Procedure: The study was conducted in the contrast laboratory of the Fukuoka Dental College Hospital. In the room was a physician who performed fluoroscopy, a speech-language-hearing therapist (SLHT) responsible for checking the position of Nodomiru’s measurement module and initiating measurements via the laptop software, and the participant. A lead ball (11 mm in diameter) was taped to the participant’s lower jaw, and he was seated in front of a fluoroscopy device (DREX-U180, CANON, Tokyo, Japan) while wearing protective gear. The software was launched on a laptop in preparation for measurement. The instrument’s sensor array was placed vertically in the midline of the participant’s neck relative to the larynx. The instrument was held by the participant with one hand, with a posture that clarified the larynx by guiding his gaze slightly upward (Figure 2). Head and neck movements were suppressed by pressing the instrument against the neck while the elbow rested on a desk. The microphone used in VF was attached to the participant’s chest, serving as a temporal marker by detecting the buzzing sound emitted by Nodomiru.

After confirming that the physician had initiated fluoroscopy, the SLHT pressed the measurement button in the software, and the participant was instructed to swallow saliva immediately after hearing a 0.5-second buzzing sound emitted from Nodomiru, signifying that measurement had started. The measurement duration was 5 seconds, and the participant was directed not to move their head or neck during the measurement. The SLHT also advised the participant to preload saliva on their tongue. The participant executed a single saliva swallow. The Nodomiru software recorded data output every 100 frames/s once the analyze button was pressed. VF images were recorded at 30 frames/s using a fluoroscopy-linked recorder (HVR-1000A, Hibino, Tokyo, Japan).

**Figure 3:**
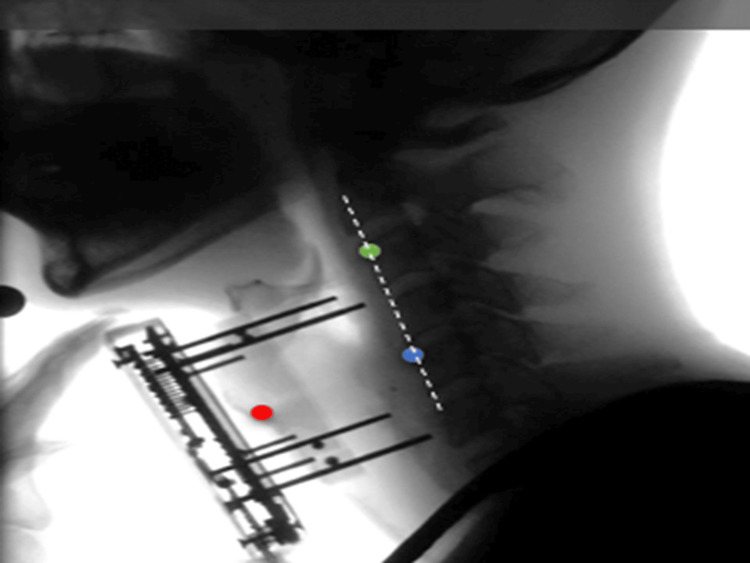
Measurement points during VF Measurement points included the superior border of the anterior commissure of the vocal folds (red circle), the anterior superior edge of the third cervical vertebra (yellow-green circle), and the anterior inferior edge of the fifth cervical vertebra (blue circle). VF, videofluoroscopy

Analysis methods: The VF recordings were analyzed using Dipp Motion V (DITECT, Tokyo, Japan), motion analysis software that automatically tracks measurement points, draws graphs, and outputs data points. Measurement points included the superior border of the anterior commissure of the vocal folds, the anterior superior edge of the third cervical vertebra, and the anterior inferior edge of the fifth cervical vertebra (Figure 3). The automatic tracking function captured the anterior upper end of the third cervical vertebra and the anterior lower end of the fifth cervical vertebra. Owing to automatic tracking challenges, manual plotting of arbitrary frames was employed for the superior border of the anterior commissure of the vocal folds using the modified tracking function. The automatic tracking of the anterior border of the anterior commissure of the vocal cords tends to be incomplete due to the rapid up-and-down movement involved. For this reason, the SLHT had to fine-tune the plot to an arbitrary position based on the image of one frame. The reference axis, Y axis, and X axis were set as the origin at the anterior lower end of the fifth cervical vertebra, a straight line connecting the anterior upper end of the third cervical vertebra and the anterior lower end of the fifth cervical vertebra, and a straight line passing through the anterior lower end of the fifth cervical vertebra and orthogonal to the Y axis, respectively. For analysis, the upper border of the anterior commissure of the vocal folds was defined as larynx movement, and time-specific data were exported to an Excel sheet.

Subsequently, a moving average was calculated for each of the three datasets to apply the smoothing process performed by Nodmiru to the VF data. More specifically, if the measurement data is arranged in order as a, b, c, d, e, etc., the moving average data will be a+b+c, b+c+d, c+d+e, etc. Given that VF and Nodomiru record at 30 frames/s (30 Hz) and 100 frames/s (100 Hz, f1, Wave 1), respectively, their sampling rates differ. Therefore, the VF data were resampled. Resampling is the process of setting a new time interval and supplementing it. Specifically, the least common multiples of 30 and 100 Hz were used to unsample the VF data (30 Hz, f2, Wave 2) to 300 Hz (f3, Wave 3), filling missing data with “0.” A low-pass filter of f1/2 Hz was then applied to Wave 3 to smooth the waveform where it was complemented with “0.” Additionally, Wave 3 was adjusted to the original waveform level by multiplying it by f3/f2, and resampled data (100 Hz, f4, Wave 4) were generated by thinning out every f3/f1 point from Wave 3.

To determine the end of Nodomiru’s 0.5-second buzzing sound, the audio waveform was checked using the audio editing function of Video Proc Blogger (Chengdu Digiarty Software, Sichuan, China), and the amplitude disappearance point was identified to estimate the start of the measurement by Nodomiru. Thus, Nodomiru’s start timing was determined by identifying the amplitude disappearance position.

By having SLHT A analyze the Nodomiru data and SLHT B analyze the VF data, both were blinded.

Nodomiru and VF output data were depicted as a laryngeal elevation curve in a single graph. The VF microphone picking up Nodomiru’s buzzing sound introduced a time delay in the VF data. To resolve this issue, in the laryngeal movement curve generated from Nodomiru and VF data, the point just before the onset of laryngeal movement was identified and corrected for temporal deviation.

Statistical analysis: Correlation coefficients for data points over time were calculated for upward-downward and anterior-posterior movement in both Nodomiru and VF data to verify the validity of the Nodomiru measurement. IBM SPSS Statistics for Windows, Version 25.0 (Released 2017; IBM Corp., Armonk, NY, USA) was used to perform statistical analysis, with p < 0.05 considered statistically significant.

Step 2: Intrarater Reliability of Peak Velocity and Laryngeal Movement Distance Measured Using Nodomiru

The present study’s dataset partially overlapped with that of our previous study [17]. Inclusion criteria comprised being a male aged 20-39 years or a male aged 60-79 years. Exclusion criteria included a history of cerebrovascular disease, neurodegenerative disease, or head and neck cancer, the presence of obvious dysphagia (e.g., coughing with swallowing), and a participant scoring ≥3 points on the Eating Assessment Tool-10 (EAT-10). To recruit healthy young participants, male staff members aged 20-39 years were individually contacted at the hospital where the principal investigator works. Healthy older men were recruited from registered members of the Silver Human Resource Center through an administrator at the center who understood the study’s purpose. Inclusion/exclusion criteria, excluding the EAT-10 score, were checked by the administrator, with the principal investigator confirming the EAT-10 score during the participant’s initial visit to the research lab. Medical history was also verified by the principal investigator.

At the study’s outset, participants included 21 healthy young adult men aged 20-39 years (mean: 28.3 ± 7.2 years; range: 20-38 years) and 22 healthy older men aged 60-79 years (mean: 73.3 ± 3.5 years; range: 66-79 years). None of the participants exhibited a history of diseases potentially affecting swallowing, e.g., anterior neck surgery, reflux disease, obstructive sleep apnea, pulmonary disease, or cardiovascular surgery. Participants received a written explanation of the study, and each participant provided their written consent to participate.

**Figure 4:**
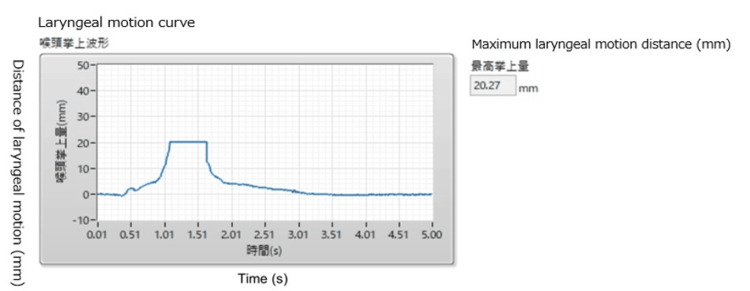
Screen display on Nodomiru immediately after measurement

Measurement methods: The Speech Therapy Room, Fukuoka Dental College Hospital, served as the measurement site, with one SLHT and one participant assigned, and measurements performed on one person. First, the software was launched from a laptop in preparation for measurement. The instrument’s sensor array was placed vertically in the midline of the participant’s neck relative to the larynx. The instrument was held by the participant with one hand, with a posture that clarified the larynx by guiding his gaze slightly upward. Head and neck movements were suppressed by pressing the instrument against the neck while the elbow rested on a desk. When the measurement button was pressed in the Nodomiru software installed on a notebook PC, a 5-second measurement began after a 0.5-second buzzer sound. The participants were instructed to swallow the saliva that had been collected on their tongues. They were also instructed not to move their heads or necks during the measurement. Upon measurement completion, analysis results were promptly displayed by pressing the analysis button (Figure 4). Each participant was asked to swallow saliva five times, with a one-minute break allotted between administrations to prevent fatigue. Participants were allowed to drink a minimum amount of water during breaks to avoid dry mouth. However, they were repeatedly instructed to only swallow saliva during the measurement.

Statistical analysis: The required sample size was calculated as 16.84, using the formula 3.5 = 1.96 × 7.33/√n when the margin of error was set at 3.5 and the SD at 7.33.

Intraclass correlation coefficients (ICCs) were calculated to determine the intraclass reliability of peak velocity and laryngeal movement distance for upward-downward and anterior-posterior movement. For intraclass reliability, ICC (1,1) and ICC (1,5), estimating the reliability of the average of five measurements, were obtained. In Nodomiru’s measurement, the examiner only presses the start button and instructs the participant to swallow, so there is little possibility of differences in measurement between examiners. For this reason, we only examined the intrarater reliability.

## Results

Step 1: Validity of the laryngeal movement curve generated using Nodomiru

**Figure 5:**
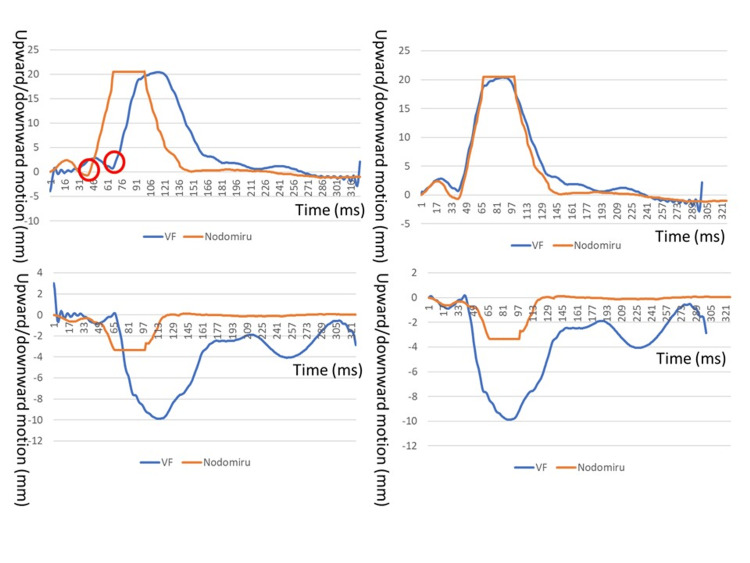
Laryngeal movement curves generated by Nodomiru and VF (a) and (b) represent the upward-downward movement of the larynx, while (c) and (d) represent the anterior-posterior movement. (a) Points just preceding the start of the laryngeal movement were identified, and a temporal deviation of 0.27 seconds was confirmed (red circles). (b) After correcting for the misalignment, nearly identical laryngeal movement curves were obtained. Anterior-posterior movement curve (c) before and (d) after temporal correction. VF, videofluoroscopy

Examining the laryngeal movement curves for upward-downward movement generated by Nodomiru and VF revealed a temporal deviation of 0.27 seconds just before the onset of laryngeal movement (Figure 5a). When the VF data was subtracted by 0.27 seconds and the starting point was set to 0 mm, an almost identical laryngeal movement curve was drawn (Figure 5b). The maximum laryngeal movement distances were 20.5 and 20.4 mm for Nodomiru and VF, respectively, demonstrating close agreement. Conversely, for anterior-posterior movement, even after correcting for the 0.27-second discrepancy, no matching laryngeal movement curve was obtained (Figure 5c, 5d). The maximum laryngeal movement distance was −3.40 and −9.98 mm for Nodomiru and VF, respectively, indicating a clear difference in values.

The correlation coefficient between the hourly data points for upward-downward movement obtained using Nodomiru and those obtained after correcting for the temporal shift in VF was r = 0.989 (p < 0.01), signifying a high correlation. Similarly, the correlation coefficient for anterior-posterior movement was high at r = 0.834 (p < 0.01).

Step 2: Intrarater reliability of peak velocity and laryngeal movement distance measured using Nodomiru

The laryngeal elevation curve could not be drawn for two of the 21 young men and three of the 22 elderly men. When the larynxes of the five men for whom the laryngeal elevation curve could not be drawn were visually checked, it was difficult to judge their positions from their external appearance alone. As the laryngeal elevation curve could not be drawn, these participants were excluded from the results.

Peak velocity of laryngeal movement: For upward-downward laryngeal movement, the average peak velocity was 165.0 ± 78.7 mm/s for young males and 171.1 ± 96.1 mm/s for older males. The ICC (1,1) for the upward-downward movement was 0.779 for young men, 0.822 for older men, and 0.801 overall, whereas the ICC (1,5) was 0.946 for young men, 0.959 for older men, and 0.959 overall (Table 1). The anterior-posterior laryngeal movement had an average velocity of 36.0 ± 32.7 mm/s for younger men and 28.9 ± 27.7 mm/s for older men. The ICC (1,1) for the anterior-posterior movement was 0.839 for younger men, 0.598 for older men, and 0.737 overall, whereas the ICC (1,5) was 0.963 for younger men, 0.881 for older men, and 0.933 overall (Table 1).

**Table 1:** ICCs for laryngeal movement ICC, intraclass correlation coefficient

Measurement	Direction	Young adult men		Older men		Overall	
		ICC (1,1)	ICC (1,5)	ICC (1,1)	ICC (1,5)	ICC (1,1)	ICC (1,5)
Peak velocity	Upward-downward	0.779	0.946	0.822	0.959	0.801	0.953
	Anterior-posterior	0.839	0.963	0.598	0.881	0.737	0.933
Distance	Upward-downward	0.638	0.898	0.772	0.944	0.705	0.923
	Anterior-posterior	0.855	0.967	0.312	0.694	0.779	0.946

Laryngeal movement distance: The average upward-downward laryngeal movement distance was 22.8 ± 7.0 mm for younger males and 23.6 ± 7.6 mm for older males. The ICC (1,1) for the upward-downward movement was 0.638 for young men, 0.772 for older men, and 0.705 overall, whereas the ICC (1,5) was 0.898 for young men, 0.944 for older men, and 0.923 overall (Table 1). The average anterior-posterior laryngeal movement distance was 1.9 ± 2.3 mm for younger men and 1.0 ± 1.0 mm for older men. The ICC (1,1) for the anterior-posterior movement was 0.855 for younger men, 0.312 for older men, and 0.779 overall, whereas the ICC (1,5) was 0.967 for younger men, 0.694 for older men, and 0.946 overall (Table 1).

## Discussion

Step 1: Validity of the laryngeal movement curve generated using Nodomiru

The laryngeal movement curves generated by VF and Nodomiru for upward-downward movement were almost identical. Correspondingly, the correlation coefficients between the respective data points were also high. It was found that Nodomiru can easily and noninvasively measure the upward-downward movement of the larynx of healthy men. Conversely, no distance agreement emerged for measurements of the anterior-posterior movement. The man in his 30s in Step 1 was thin and had a clear laryngeal contour, but there was a clear difference in distance between VF and Nodomiru in the anterior-posterior movement, so we suspect that there is a problem with the sensitivity itself. Haji [16] also acknowledged this limitation in the system, emphasizing the need for further improvement, which is supported by the results of our study. Nevertheless, the present results partially support our hypothesis that laryngeal movement curves generated using VF align with those produced by Nodomiru, specifically for upward-downward movement.

Several attempts have been made to noninvasively measure laryngeal movement during swallowing [12,13], notably using the piezoelectric pressure sensor [14,15] and B4S™ [13]. These attempts aim to facilitate the acquisition of swallowing maneuvers, which involve voluntary adjustments to specific swallowing movements. The objective is to modify an individual’s swallowing maneuver, ultimately promoting safer swallowing. Among these maneuvers, mastering the Mendelssohn maneuver often requires dedicated practice. This voluntary adjustment of laryngeal movement aims to increase the duration and extent of laryngeal elevation, thereby promoting greater extension of the esophageal sphincter opening over a longer period [18-22]. Hence, measuring both the duration and distance of laryngeal movement and providing feedback are essential for accurate Mendelssohn maneuver execution. Notably, Nodomiru is considered superior to previous devices as it enables feedback on both laryngeal movement distance and duration.

It is important to emphasize that the participants of this study were limited to healthy men. The comparison with VF was conducted only to verify the validity of the laryngeal elevation measurement. Needless to say, Nodomiru can only measure laryngeal elevation movement, so it cannot be used as a substitute for VF. At present, the applicability of Nodomiru is limited to elderly men without dysphagia who do not require VF-based evaluation, such as those participating in care prevention programs.

Step 2: Intrarater reliability of peak velocity and laryngeal movement distance measured using Nodomiru

For upward-downward movement, both peak velocity and laryngeal movement distance exhibited robust intrarater reliability in all participants, with enhanced accuracy noted when the average of five measurements was considered. In recent years, several studies have analyzed the peak velocity of the hyoid and larynx, considering the characteristics of hyoid and laryngeal movement during swallowing [1-3,23-25]. Laryngeal peak velocity has received particular attention owing to its association with more severe dysphagia [1] and its utility in predicting laryngeal penetration and aspiration [2]. In the fields of motor control and movement kinematics, peak velocity or speed measurements are often interpreted as indicators of muscular power [26-28]. Specifically, the peak velocity of the hyoid and larynx are thought to reflect the muscular power of the supralaryngeal muscle group [1]. Thus, Nodomiru’s capability to accurately measure peak velocity, albeit limited to upward-downward movement, is a notable strength. In the future, it is hoped that this system will be used not only for visual feedback during training but also for assessing the risk of aspiration after reliability and validity have been verified in patients with dysphagia.

The intrarater reliability for the anterior-posterior movement was low for elderly men. This suggests that, in addition to the sensitivity issues indicated in Step 1, problems specific to elderly men, such as sagging skin, may be affecting the measurement of anteroposterior movement.

Molfenter and Steele [29], in a comprehensive literature review of hyoid and laryngeal displacement in healthy populations, identified concerns about the limited number of swallows per subject in many studies. In seven of the reviewed studies, only two swallows per bolus condition were analyzed, and other studies restricted the analysis to a single swallow per bolus condition [7-10]. Lof and Robbins [30] recommend that at least three repeated swallowing trials be included in the VF at each bolus dose to balance the need to capture individual differences with the adverse effects of radiation exposure. Additionally, Molfenter and Steele [29] suggested that future studies should maximize both the number of participants and swallows per condition to reduce variability. Given Nodomiru’s radiation-free nature, we recommend performing measurements under the same conditions approximately five times to improve reliability.

In terms of laryngeal movement distance, Molfenter and Steele [29] reported a range of 21.1 mm [11] to 33.9 mm [6] across the reviewed studies for displacement of the superior larynx. This wide variability among studies was influenced by bolus volumes, with higher volumes tending to result in greater displacement. Therefore, the present results are reasonable, given that the mean laryngeal movement distances for younger and older men were 22.8 and 23.6 mm, respectively, and that high validity was observed for Step 1.

The literature review by Molfenter and Steele [29] also indicated anterior laryngeal displacement ranging from 3.4 mm [4] to 8.2 mm [5,6]. This motion exhibited less variability among studies compared with anterior hyoid displacement, with a trend toward greater displacement observed with higher bolus volumes and a wide range of values noted across studies. In the present study, the mean anterior displacement was 1.9 mm for younger males and 1.0 mm for older males, notably lower than reported in previous studies. Despite accounting for low bolus volume, the values in the current study were considerably lower, suggesting a potential limitation in Nodomiru’s sensitivity to anterior-posterior movement. Nevertheless, the findings from Step 2 partially support our second hypothesis that the intrarater reliability of peak velocity and laryngeal movement distance measured by Nodomiru is high, specifically for upward-downward movement but not an anterior-posterior movement.

Limitations

This study is limited by its exclusive focus on men, as the studied system’s external laryngeal movement detection leads to difficulties when recording the larynx in women, particularly those with an obscured larynx or pronounced obesity. Although Haji [16] suggests that laryngeal movement could be measured in women with a visible larynx, further investigation is required to determine the system’s effectiveness in female subjects. Additional research will also be needed to determine the effects of BMI and laryngeal visibility on Nodomiru measurements. Furthermore, the need for a slightly extended neck position to enhance laryngeal visibility poses a limitation. Consequently, caution is advised against using this system in severely dysphagic patients, those prone to aspiration, or those who would aspirate without compensatory head and neck movements.

This study has not been conducted on patients with dysphagia. Therefore, it is unclear whether the Nodomiru can correctly detect laryngeal elevation in patients with dysphagia, who are more likely to have variable results. In the future, it will be necessary to conduct new verification studies on patients with dysphagia. However, since its reliability has been demonstrated in healthy older men, it is thought that it can be used for evaluating swallowing and determining the effects before and after intervention in nursing care prevention projects.

Step 1 only involved one healthy young adult man, due to the effects of X-ray exposure. Further validation is required for multiple subjects in studies involving patients with dysphagia.

## Conclusions

Nodomiru showed high agreement with VF in measuring laryngeal movement in the upward-downward movement. In addition, high interrater reliability was confirmed when measuring peak velocity and elevation distance in upward-downward movement. On the other hand, improvements are needed to improve the accuracy of measurements in the anterior-posterior movement. Nodomiru is a useful tool for quickly and noninvasively evaluating laryngeal elevation, and it is expected to have clinical applications such as visual and objective feedback for training.
